# The level of spiritual care competence of Polish nurses and the psychometric properties of the spiritual care competence scale (SCCS)

**DOI:** 10.1186/s12912-022-00889-z

**Published:** 2022-05-06

**Authors:** Michał Machul, René van Leeuwen, Dorota Ozga, Krzysztof Jurek, Sylwia Boczkowska, Beata Dobrowolska

**Affiliations:** 1 Department of Holistic Care and Management in Nursing , Faculty of Health Sciences, Medical University of Lublin, Staszica Str. 4-6, Lublin, Poland; 2 Faculty of Health Care, Viaa University of Applied Sciences Zwolle, Zwolle, Netherlands; 3 Institute of Health Science, University of Rzeszów, Rzeszów, Poland; 4 Institute of Sociology, Faculty of Social Sciences, John Paul II Catholic University, Lublin, Poland

**Keywords:** Spiritual care, Competence, Spiritual care competence scale, Psychometric properties, Nurses

## Abstract

**Background:**

Providing effective spiritual nursing care requires development of professional competence which, when regularly evaluated, allows one to direct undergraduate and postgraduate nursing education in order to develop knowledge, skills and attitudes of nurses in the scope of spiritual care.

**Aim:**

The aim of this study was to analyse the level of spiritual competence of professionally active nurses in Poland and, additionally, to analyse the psychometric properties of the Spiritual Care Competence Scale (SCCS).

**Methods:**

A cross-sectional study among Polish nurses (*n* = 343) was performed in accordance with the STrengthening the Reporting of OBservational studies in Epidemiology guidelines.

**Results:**

An exploratory factor analysis identified five factors with 27 items explaining a total variance of 64.75%. Cronbach’s alpha coefficient for the subscales ranged from 0.70 for ‘Attitude toward the patient’s spirituality’ to 0.92 for ‘Professionalisation and improving the quality of spiritual care’. Nurses reported a high level of spiritual competence (104.39 points) with better results in ‘Attitude toward the patient’s spirituality’ and ‘Communication, personal support and patient counselling’ than in the ‘Assessment and implementation of spiritual care’, ‘Professionalisation and improving the quality of spiritual care’, and ‘Referral, consultation and spiritual care’. Significant correlation was found between nurses’ age, job seniority and spiritual competence, and between religiosity and spiritual competence.

**Conclusions:**

The study showed satisfactory psychometric properties of the Polish version of the Spiritual Care Competence Scale, confirming its potential to measure the level of spiritual competence of nurses, both in education and research processes. SCCS-PL revealed five-factor structure with good internal consistency. The findings highlight the importance of providing professional education in respect of spiritual nursing care, especially in its practical dimension regarding skills development in which nurses obtained lower scores.

## Introduction

Spirituality is a multidimensional phenomenon embedded in the very essence of human existence and in how people discover who they actually are [1]. It permeates all forms and areas of existence – relationships, everyday life; it drives the search for higher values and for what gives us a sense of meaning. Therefore, spiritual well-being is a fundamental indicator of health [2]. Encompassing much more than just physical fitness, it includes one’s emotions and feelings, the sense of integration with themselves and the world around them [3]. Spirituality and religious beliefs influence the way people cope with illness and stressful situations in life, whereas religious or spiritual practices can significantly contribute to the quality of life as well as promote health behaviours [4] and provide social support. On the other hand, they can induce anxiety and augment the sense of illness [5].

Reports from practitioners and published literature clearly indicate that spirituality should be integrated into nursing care [6] and that its marginalisation can negatively impact on the quality of care [7]. An assessment of spiritual health seems to be as necessary as physical or psychosocial assessment [8]. A comprehensive care plan should include a holistic approach to the patient, taking into account their spiritual needs. Many studies have shown that medical staff providing spiritual care to patients substantially contributes to their overall well-being, which has a positive impact on the human immune functions [9–12]. Having nursing staff include spirituality in their care plans can help patients find hope and meaning in the course of an illness or crisis. Nurses, on the other hand, may find that spirituality helps them find meaning and purpose in their work. Therefore, the authors of many publications argue that nursing should address spirituality in its clinical practice [13–15].

Spiritual care is an integral part of holistic nursing [16]. In literature it is defined in many ways [17–19]. It goes beyond just psychosocial care - it means helping to acquire and maintain all existential aspects of a patient’s life through meaning, presence, support or care-oriented interventions. All of this is aimed at helping the patient find meaning and purpose in the disease, throughout his life, as well as ensure well-being by achieving inner peace and a new perspective on himself [20]. The aim of spiritual care is to assess and meet the spiritual needs of patients in close collaboration with members of an interdisciplinary treatment team [21, 22]. Spiritual care becomes a response to spiritual needs, it is a matter of recognizing what patients are not receiving [19]. Issues of spirituality and its nature in nursing are linked to the concept of competence in spiritual care, defined as the knowledge, skills and attitudes required to provide spiritual care [23]. Factors that may contribute to its development include personal life experiences [24], personal spirituality [25] and professional work. Integrated educational programmes and developed standards for the implementation of spiritual care are important and must take into account not only theoretical but also clinical aspects [26, 27]. In consequence, nurses define spirituality more precisely [28]; they also have considerable self-awareness about personal spirituality [29] and thus also exhibit a greater capacity to provide spiritual care [30].

COVID-19 pandemic has posed new challenges for healthcare around the world [31]. Some activities on the part of chaplains and the opportunity to pursue spiritual practices have been significantly reduced in order to control infection in hospital wards [31]. Due to the increased sanitary regime and the ban on visits to medical facilities, coronavirus-infected patients have been isolated from their families. Greater isolation has contributed to a more pronounced sense of loneliness and reduced quality of life [32]. These experiences show that it is necessary to develop the competence to implement spiritual care among medical personnel, including nurses, who are closest to and spend most time around the patient.

There are several tools allowing the assessment of nurses’ competence to provide spiritual care, e.g. Spiritual and Religious Care Competencies for Specialist Palliative Care [33], Spiritual Care Competence Questionnaire [34], Nurses’ Spirituality and Delivery of Spiritual Care [35], and Spirituality and Spiritual Care Rating Scale [14]. One of them is the Spiritual Care Competence Scale (SCCS) developed by van Leeuwen and colleagues [23]. The tool was selected deliberately, it is a reliable tool with good internal consistency and appropriate correlations between items. Additionally, it has been often applied in international studies [36]. In Poland, nurses’ competencies in this area have not been assessed and there is no tool available in Polish to study this aspect of nursing practice and thus to influence nursing undergraduate and postgraduate education, as well as to undertake international studies and comparative analyses. Consequently, the present study aims to analyse the level of spiritual competence of professionally active Polish nurses and the psychometric properties of the SCCS.

## Methods

### Aim

The aim of the research is to analyse the psychometric properties of the SCCS and, additionally, to analyse the level of spiritual competence of professionally active nurses in Poland.

### Study design

A cross-sectional study performed in 2019 in accordance with the STrengthening the Reporting of OBservational studies in Epidemiology (STROBE) guidelines [37].

### Study participants

Convenience sampling method was applied and the study was conducted among nurses employed in healthcare institutions (i.e. hospital, hospice, outpatient clinic) in two neighbouring provinces of eastern Poland. The inclusion criteria for the study were the nurse’s consent to fill in the questionnaire, being professionally active (i.e. currently employed as a nurse), and to have at least 1 year of working experience as a RN. In the second phase of the study, for evaluation of the psychometric properties of the SCCS – Polish version, 392 questionnaires were distributed and 345 responses were received, which means a 0.88% return rate. A total of 343 participants who completed the questionnaire correctly were included in this analysis.

### Instruments

Three instruments were used in the study:

- The Spiritual Care Competence Scale (SCCS), as developed by van Leeuwen et al. [23]. It includes the following six subscales: *Assessment and implementation of spiritual care; Professionalisation and improving the quality of spiritual care*; *Personal support and patient counselling*; *Referral to professionals*; *Attitude toward the patient’s spirituality*; and *Communication*. The scale consists of 27 questions, scored on a Likert-type scale: From “strongly disagree” (1) to “strongly agree” (5). The minimum and maximum possible values are 27 and 135, respectively. When calculating the nurses’ scores received in SCCS, a minimum score of 27 and a maximum score of 135 were considered. The score lower than 64 indicates low spiritual competence, the score of 64–98 indicates average spiritual competence, and the score of 99 and above implies high spiritual competence [38]. The Cronbach’s alpha value for the subscales ranges from 0.56 for *Attitude toward the patient’s spirituality* to 0.82 for *Assessment and implementation of spiritual care* and *Professionalisation and improving the quality of spiritual care* [23]. Written consent was obtained from the author of the scale to use it in the process of validation.
  The process of cultural adaptation and translation of the SCCS, performed in line with the International Test Commission guidelines [39], consisted of several stages: Two bilingual translators whose mother tongue is Polish and who are fluent in the source text language prepared two independent translations (from English into Polish) of the scale. At the next stage, the results of their work were synthesised – both versions were brought together and merged into one translation. A preliminary version of the scale in Polish was presented to five Competent Judges (a nursing student, a unit nurse, a ward nurse, a theologian/clergyman, and a university researcher specialising in spirituality) to evaluate the comprehensibility, communicability and unambiguity of the translated scale questions. Having been prepared in accordance with Competent Judges’ suggestions, the scale was back-translated by a native speaker of English – a university teacher who has lived in Poland for many years. The grammatical form of the statements and categories of responses, their number and order of occurrence, and the instructions for conducting the study, taking into account the selection of the sample were kept as is. Next, the Polish version of the tool was pre-tested in a group of ten nurses and all their comments were incorporated into the final version of the scale in Polish.
- The Duke University Religion Index (DUREL) in its Polish version [40]. The scale measures religious commitment and consists of 5 questions divided into three parts: Organised religious activity (ORA), non-organised religious activity (NORA), and intrinsic religiosity (IR). The Polish version of the scale is characterised by Cronbach’s alpha index of 0.91.
- A short form collecting sociodemographic characteristics such as: Age, sex, marital status, place of residence, job seniority, education, religious affiliation, and the place of work.

### Data collection

The study was conducted in two phases (test-retest) between March and the end of May 2018. The retest took place on average 3 weeks after the test. When planning the sample power, the ratio 1:10 (one item per 10 study participants) was considered [41], which in our case means a minimum of 270 respondents. Taking into account the cases of refusal and withdrawal from participation in the survey, in the test phase 392 questionnaires were distributed and 345 responses were received, which means a 0.88% return rate. Three hundred forty-three forms (0.99%) were found eligible for statistical analysis. In the retest phase 77 fully completed questionnaires were returned. The limited number of retest participants is largely a consequence of missing information that would allow appropriate coding of the questionnaires and pairing of the test and retest forms.

The questionnaires were delivered to postgraduate training centres, where local nurses undergo professional training, and to healthcare facilities. The request to assist in the distribution of the questionnaires was addressed to the heads of postgraduate training centres and to the chief nurses of healthcare facilities. The researcher explained to the respondents the purpose and significance of the study and informed them that participation was completely voluntary. Envelopes with information about the study, an informed consent form, and the questionnaire were handed out to all the respondents who were asked to deliver them, after filling in, to the office of the head of a postgraduate training centre or the chief nurse of the healthcare facility.

### Statistical analysis

The collected research material was statistically processed using the IBM SPSS Statistics (PS IMAGO) suite in its latest version v.25.

Cronbach’s alpha coefficient was used to assess reliability by testing the internal consistency of the scale. The adequacy of sampling was examined using the Kaiser-Meyer-Olkin test – it is a coefficient which compares partial correlations with bivariate correlation coefficients. Theoretical relevance was assessed using exploratory factor analysis conducted with the use of the principal components method which employed simple Oblimin rotation with Kaiser normalisation. The reliability of the tool was estimated on the basis of the discriminatory power values of the items forming the distinguished dimensions. Correlations between the variables, depending on the level of measurement of the variables, were assessed with the Pearson’s r or Spearman’s rho coefficient. Among reliability analyses, the most important are internal consistency, interrater reliability, and test-retest reliability. Test-retest reliability can be defined as “a measure of the reproducibility of the scale, that is, the ability to provide consistent scores over time in a stable population. Many factors can influence the results at different times, eg respondents may have different moods, or external conditions may influence their ability to react [42]. The smaller the difference between the two sets of results, the higher the test-retest reliability. The stability of the tool SCCS (test-retest) was checked using a t-test. A significance level of *p* < 0.05 was adopted, indicating the presence of statistically significant differences or relationships.

The study group from the analysis of data derived from responses to the questions in the metric was characterised using the following measures of location and dispersion: Structure index, median, arithmetic mean, measures of dispersion, standard deviation.

### Ethical issues

A positive opinion of the Bioethics Committee of the Medical University of Lublin number KE-0254/285/2018 dated 29 November 2018 was obtained to conduct the study. Nurses were invited to the study on a voluntary basis. All respondents were advised about the course of the research and the principle of confidentiality and anonymity during the research process.

Every respondent was given two envelopes: One for signed informed consent for taking part in the study and the second one for the completed questionnaire. This approach was adopted to respect anonymity of the respondents. Additionally, all study participants received information about the possibility of withdrawing from the study at any time. In order to pair the questionnaires for the test-retest analysis, a coding system was adopted to ensure the anonymity of the respondents [43].

## Results

### Study participants

The study comprised 343 professionally active nurses, the majority of whom were women (91%). The mean age of the respondents was 36 years and the average job seniority was 13 years. The vast majority of the respondents declared to be Catholics (95.6%) and 52.8% of respondents were married. Most of the nurses held the master’s (47,3%) and bachelor’s degree in nursing (41.7%). They worked in surgical wards (27.1%), medical treatment wards (25.9%), and Intensive Care Units (21.0%) (Table 1).

**Table 1:** Characteristic of study participants

Socio-demographic variables		Test [*n* = 342]		Retest [*n* = 77]	
		M	SD	M	SD
Age		36,35	10,08	39,31	10,29
Job seniority		13,21	10,47	15,61	11,42
		N	%	N	%
Sex	Male	312	91.0	71	92.2
	Female	31	9.0	6	7.8
Place of residence	Urban area	210	61.2	46	59.7
	Rural area	133	38.8	31	40.3
Education	Medical Secondary School	38	11.1	6	7.8
	Bachelor’s degree in nursing	143	41.7	24	31.2
	Master’s degree in nursing	162	47.3	47	61.0
Place of work	Medical treatment ward	89	25.9	25	32.5
	Surgical ward	93	27.1	20	26.0
	ICU	72	21.0	15	19.5
	Ward without beds	20	5.8	3	3.9
	Outpatient clinic	33	9.6	9	11.7
	Hospice	22	6.4	1	1.3
	Other	21	6.1	4	5.2
Religious affiliation	Catholicism	328	95.6	74	96.1
	Protestantism	1	0.3	0	0.0
	Orthodoxy	1	0.3	0	0.0
	Other	1	0.3	1	1.3
	Nonbeliever	12	3.5	2	2.6
Job satisfaction	Definitely yes	150	43.7	29	37.7
	Rather yes	159	46.4	37	48.1
	Hard to say	22	6.4	9	11.7
	Rather no	10	2.9	2	2.6
	Definitely no	2	0.6	0	0.0

### Exploratory factor analysis

The 6-factor solution, consistent with the original version, proved unsatisfactory. This solution explained 68.19% of the variability. The sixth factor, however, had an eigenvalue of less than 1.

With an unforced number of factors, 5 eigenvalues above 1.0 were obtained. Taken together, they explain 64.75% of the variability. The value of the Kaiser-Meyer-Olkin sampling adequacy test is 0.933 and the Bartlett sphericity test yielded a significant result (χ2 = 5722.127; df = 351; *p* < 0.001). The factor analysis satisfies the Kaiser criterion (three loadings above the value of one). The analyses conducted revealed, in most cases, moderate to high values of the factor loadings of all the items forming the individual subscales. Several items displayed high loadings in two factors (items 1, 13, 5, 6, 19, 20, 21). However, they were assigned to the factors that were obtained in the original version of the tool (Table 2).

**Table 2:** Explorative factor analysis and internal consistency of SCCS-Pl (*n* = 343)

Items of SCCS (*)	Factors, items loading				
	Factor 1	Factor 2	Factor 3	Factor 4	Factor 5
Item 1	0.650		0.338		
Item 2	0.624				
Item 3	0.767				
Item 4	0.596				
Item 7		0.468			
Item 8		0.496			
Item 9		0.628			
Item 10		0.715			
Item 11		0.764			
Item 12		0.699			
Item 13		0.495	0.392		
Item 14			0.707		
Item 15			0.776		
Item 16			0.636		
Item 17			0.517		
Item 5		0.352		0.607	
Item 6		0.362		0.567	
Item 18				0.567	
Item 19				0.441	0.534
Item 20				0.487	0.524
Item 21			0.423	0.447	
Item 22					0.617
Item 23					0.812
Item 24					0.728
Item 25					0.788
Item 26					0.780
Item 27					0.817
Eigenvalue	2.32	11.40	1.02	1.44	1.30
Variance %	8.60%	42.18%	3.79%	5.34%	4.80%

Legend: *SCCS* Spiritual Care Competence Scale

(*) item numbers are coming from original version of the SCCS

Factor 1 - Attitude toward the patient’s spirituality; Factor 2 - Assessment and implementation of spiritual care; Factor 3 - Referral, consultation and spiritual care; Factor 4 - Communication, individual support and counselling; Factor 5 - Professionalisation and improvement of the quality of spiritual care

Statistical analysis allowed the replication of the three original factors: (1) ‘Attitude toward the patient’s spirituality’, (2) ‘Assessment and implementation of spiritual care’, (5) ‘Professionalisation and improving the quality of spiritual care’. Three other factors from the original version of the SCCS were used to create two new factors: (4) ‘Communication, personal support and patient counselling’, (3) ‘Referral, consultation and spiritual care’.

### Reliability

The value of Cronbach’s alpha coefficient for the entire scale is 0.95. The factor with the lowest α value was ‘Attitude toward the patient’s spirituality’ (= 0.70), while the highest α value was reported for factor ‘Assessment and implementation of spiritual care’ (=0.92) and ‘Professionalisation and improving the quality of spiritual care’ (=0.92). The correlations between the scales and the overall result were statistically significant. The reliability of the scale was evaluated using the repeated-measures test-retest method with a t-test. A group of 77 people were tested twice with the same measurement tool. This procedure showed no statistically significant differences (Table 3).

**Table 3:** Reliability of the of SCCS-Pl (*n* = 343)

Subscales of SCCS	[1]	[2]	[3]	[4]	[5]	[6]
Attitude toward the patient’s spirituality [1]	–					
Assessment and implementation of spiritual care [2]	.399^***^	–				
Referral, consultation and spiritual care [3]	.424^***^	.712^***^	–			
Communication, individual support and counselling [4]	.440^***^	.615^***^	.666^***^	–		
Professionalisation and improvement of the quality of spiritual care [5]	.306^***^	.713^***^	.624^***^	.588^***^	–	
Total score [6]	.567^***^	.889^***^	.849^***^	.816^***^	.852^***^	–
Cronbach Alpha	0.70	0.92	0.83	0.81	0.92	0.95
Test-retest [*p*-value]	0.948	0.503	0.391	0.829	0.191	0.407

*** < 0.001

### Spiritual care competence of Polish nurses

The average score obtained by Polish nurses in the Spiritual Care Competence Scale is 104.39 (SD = 15.22). It is 77.3% of the maximum point number (135). The test group obtained the highest result in *Attitude toward the patient’s spirituality*, the lowest in *Professionalisation and improving the quality of spiritual care.* Older nurses with longer job seniority received higher results in all subscales of SCCS. Higher satisfaction with work entailed higher results in all subscales of SCCS (Table 4).

**Table 4:** Polish nurses spiritual care competence and correlation with age, job seniority and satisfaction with work

Subscales of SCCS	M	SD	A	K	Min	Max	Me	Age	Job seniority	Satisfaction with work^A^
Attitude toward the patient’s spirituality	4.19	0.59	−0.90	2.13	1.00	5.00	4.25	0.120*	0.108*	−0.114*
Assessment and implementation of spiritual care	3.73	0.78	−0.55	0.39	1.00	5,00	3.83	0.149**	0.151**	−0.246**
Referral, consultation and spiritual care	3.91	0.65	−0.70	0.98	1.40	5.00	4.00	0.166**	0.180**	−0.210**
Communication, individual support and counselling	4.08	0.58	−0.74	0.99	2.00	5.00	4.00	0.116*	0.127*	−0.254**
Professionalisation and improvement of the quality of spiritual care	3.54	0.80	−0.48	0.38	1.00	5,00	3.67	0.133*	0.138*	−0.162**
Total score	104.39	15.22	−0.43	0.46	49.00	135.00	105.00	0.169**	0.174**	−0.255**

Legend: *M* mean value, *SD* standard deviation, *K* kurtosis, *A* asymmetry

^A^ 1 - definitely yes, 5 - definitely not

* < 0.05; ** < 0.01

A positive correlation was found between ORA, NORA, IR and four subscales of SCCS (besides ‘Attitude toward the patient’s spirituality’) and the total score (Table 5).

**Table 5:** Polish nurses spiritual care competence and religiosity

	PolDUREL		
Subscales of SCCS	ORA	NORA	IR
Attitude toward the patient’s spirituality	0.003	0.06	0.045
Assessment and implementation of spiritual care	0.197^***^	0.164^**^	0.277^***^
Referral, consultation and spiritual care	0.237^***^	0.139^**^	0.270^***^
Communication, individual support and counselling	0.231^***^	0.180^***^	0.302^***^
Professionalisation and improvement of the quality of spiritual care	0.213^***^	0.174^***^	0.230^***^
Total score	.567^***^	.889^***^	.849^***^

Legend: *ORA* organised religious activity, *NORA* non-organised religious activity, *IR* intrinsic religiosity

** < 0,01 *** < 0.001

## Discussion

The study analysed the level of spiritual competence of Polish nurses together with the psychometric properties of the Polish version of the Spiritual Care Competence Scale. The results showed that the psychometric properties of the Polish version of the SCCS scale are satisfactory and the scale can be used in evaluation of the nursing education in the scope of spiritual nursing care as well as in national and international research.

The Polish version of SCCS showed a five-factor structure that explains 64.75% of the variability, as opposed to the original six-factor model of the scale. The original Dutch version of the scale containing six subscales explains 53% of the total instrument variance [23], the Brazilian six-factor version explains 61.1% of the total variance; however, three items obtained factor loadings of < 0.30 [44]. The Turkish version of the SCCS revealed a three-factor structure that explains 75.18% of the variance [45].

Statistical analysis of the SCCS-PL allowed the replication of the three original factors (‘Attitude toward the patient’s spirituality’, ‘Assessment and implementation of spiritual care’, ‘Professionalisation and improving the quality of spiritual care’). Two remaining factors of SCCS-PL: ‘Referral, consultation and spiritual care’; and ‘Communication, personal support and patient counselling’ were created from three factors of the original version of the scale: ‘Communication’; ‘Personal support and patient counselling’; ‘Referral to professionals’. Comparing this structure to the Turkish three-factor model of the SCCS, only one factor remained the same as in the original tool: ‘Assessment and implementation of spiritual care’. The other two were created using the remaining five factors of the Dutch version [45].

The value of Cronbach’s alpha coefficient for the entire SCCS-PL is 0.95. The values of Cronbach’s alpha for other validations of SCCS ranged from 0.92 for Malaysian [38] and Brazilian [44] versions to 0.97 calculated for SCCS-Turkish [45]. The above scores demonstrate very high internal consistency of the tool.

All factor scores met Nunnally’s criterion: Cronbach’s alpha coefficient of > 0.7. In the case of the first factor: ‘Attitude toward the patient’s spirituality’ the value was equal 0.7. The item-total correlation values were higher than 0.3. Additionally, in all validation studies, including this current one, no differences were found between measurement 1 and measurement 2, which demonstrates the stability of the tool analysed [44, 45].

The level of spiritual care competence as calculated for Polish nurses is perceived as high (mean value of 104.39; SD = 15.22) [38]. The mean scores obtained by the nurses in the SCCS scale ranged from 3.54 (SD = 0.80) in the ‘Professionalisation and improving the quality of spiritual care’ subscale to 4.19 (SD = 0.59) in the ‘Attitude toward the patient’s spirituality’ subscale. This is an interesting result as there is no specific education in this scope (on how to professionally address patient’s spiritual needs) included neither in undergraduate nor postgraduate nursing courses in Poland. However, the national standard for nursing education includes learning outcomes related to a broad scope of social competencies (with ethical one), e.g. understanding the patient’s worldview and cultural differences, empathy in the relationship with the patient and his family, effective communication with patient and his family, and respecting patient’s rights [46]. In the study by van Leeuwen & Schep-Akkerman [47] among nurses working in hospital care, mental healthcare, and home care in the Netherlands, nurses also obtained higher scores in ‘Communication’ regarding spirituality and in ‘Attitude toward the patient’s spirituality’, as compared to ‘Professionalisation and improving the quality of spiritual care’. It seems that the attitudinal dimension of spiritual care is better developed during nursing education than the dimension related to coordination and management of spiritual care in healthcare institutions/units. This is also visible in the study among Taiwan nurses [48]. Their overall spiritual care competence level was calculated as average (mean value of 96.14), similarly to Malaysian nurses (mean value of 95.44) [38].

In our study, job seniority appears to be one of the main factors determining the level of competence in the provision of spiritual care to patients. Furthermore, in the study by Semerci et al. [6] a positive correlation was found between nurses’ seniority and their levels of spiritual care competence. The correlation between the job seniority of nurses in Poland and the level of competence of spiritual care may be the result of their clinical experience and suggests that it is at the bedside that nurses learn how to provide spiritual care. Moreover, as Semerci et al. [6] suggest, with age and working experience, nurses perceive their work in from a more holistic perspective. In the study by Chen and et al. [48], frequent provision of spiritual care was found to enhance the nurses’ spiritual care competence. Certain study results indicate that nurses provide spiritual care in a more intuitive manner and with variable frequency [49, 50]. At the same time, it is not clear what kind of competencies nurses should acquire in order to provide spiritual care and this ambiguity is often perceived as a barrier to diagnosing and addressing patients’ spiritual needs [26, 48]. Therefore, to make this important aspect of holistic nursing practice clear, emphasis should be placed on professional education in the scope of spiritual nursing care. An example of international initiative, which proposes the standard of education in this scope with a set of described and justified competencies that can be applied cross-culturally in undergraduate nursing education, is the EPICC project (*Enhancing Nurses’ and Midwives’ Competence in Providing Spiritual Care through Innovative Education and Compassionate Care*) implemented as part of the Erasmus+ programme [26]. Additionally, the monitoring and evaluation of such competencies can help identify gaps in knowledge, skills and attitudes and to set the correct direction for undergraduate and postgraduate education [44, 45].

Attitude toward spiritual care and providing spiritual care by nursing staff revealed to help them find satisfaction with work [51]. In this current study, nurses who obtained higher results in SCCS declared to have higher satisfaction with their work. This suggests that nurses who are perform their jobs on a full-time basis, including the spiritual aspect (the whole-person care), experience higher satisfaction with their profession.

A positive correlation was found between the level of spiritual care competence of Polish nurses and their religiosity as measured by PolDUREL. Results of other studies regarding the possible effect of religiosity on spiritual care competence of nurses are contradictory. In the study by Dezorzi et al. [44], those nurses who were religion-affiliated displayed significantly higher levels of spiritual care competence. In the study among Taiwan nurses, religiosity was found to be significantly associated with spiritual care competencies [48]. In contrast to these studies, Hsieh et al. [52] found that Christian/Catholic nurses had lower SCCS scores than those with no religious affiliation. In a study sample analysed by van Leeuwen & Schep-Akkerman [47] religiosity had no impact on nurses’ spiritual care competence.

### Limitations of the study

A limitation of the study stems from the fact that the scale was tested on a homogenous group of nurses who declared to be Christian. They did not represent the non-denominational population or any denomination other than Christianity in Polish society. The nurses came from only two, eastern, regions of Poland. Moreover, since SCCS is a self-reported instrument which records subjective self-assessment of nurses, this may result in response bias. Therefore, in order to obtain a more in-depth analysis into spiritual care competence of nurses, a mixed-method study is recommended, including qualitative approach. The results of the study provided initial confirmation of the reliability and relevance of the SCCS and its use as a measure of the level of spiritual care competence and factors influencing spirituality in nursing practice. However, if continued with different study samples, the testing of the SCCS would help validate its application in a multicultural society [48]. It could also aid in determining how spirituality and spiritual care are understood by various populations. It is recommended that the tool be tested among non-Christian respondents as well as across various levels of professional qualifications, including among nursing students.

## Conclusions

The study showed satisfactory psychometric properties of the Polish version of the Spiritual Care Competence Scale, confirming its potential to measure the level of spiritual competence of nurses, both in education and research processes. SCCS-PL revealed five-factor structure with good internal consistency.

While the level of spiritual competence of Polish nurses is perceived as high, those competencies which refer to assessing and addressing patients’ spiritual needs, referral and coordinating inclusion of spiritual care in healthcare institutions were self-reported as developed at a lower level. These results imply that nurses have solid foundations for providing spiritual care, but they need professional education to learn how to do it. In this context, our results echo the conclusions of other studies [6, 44, 45] underlying the importance of professional education in the scope of spiritual nursing care. This is especially important at the moment, as the coronavirus pandemic has revealed some serious flaws and the absence of an effective system of such care both in Poland and globally.

A finding of note revealed in our study is that the high level of spiritual care competencies correlates with nurses’ satisfaction with work. This points to a two-way effect of providing spiritual nursing care: Impact on the patient and on the nurses themselves. This should be taken into consideration by nursing managers and healthcare providers when planning out the provision of nursing care, organisation of work and ensuring working conditions in such a way as to provide a place for spiritual care to be delivered.

## Data Availability

All data generated or analysed during this study are included in this article.
